# Automated 2D, 2.5D, and 3D Segmentation of Coral Reef Pointclouds and Orthoprojections

**DOI:** 10.3389/frobt.2022.884317

**Published:** 2022-05-27

**Authors:** Hugh Runyan, Vid Petrovic, Clinton B. Edwards, Nicole Pedersen, Esmeralda Alcantar, Falko Kuester, Stuart A. Sandin

**Affiliations:** ^1^ Sandin Lab, Scripps Institution of Oceanography, University of California San Diego, San Diego, CA, United States; ^2^ Cultural Heritage Engineering Initiative, Qualcomm Institute, University of California San Diego, San Diego, CA, United States

**Keywords:** coral, segmentation, automated, pointcloud, orthoprojection

## Abstract

Enabled by advancing technology, coral reef researchers increasingly prefer use of image-based surveys over approaches depending solely upon *in situ* observations, interpretations, and recordings of divers. The images collected, and derivative products such as orthographic projections and 3D models, allow researchers to study a comprehensive digital twin of their field sites. Spatio-temporally located twins can be compared and annotated, enabling researchers to virtually return to sites long after they have left them. While these new data expand the variety and specificity of biological investigation that can be pursued, they have introduced the much-discussed Big Data Problem: research labs lack the human and computational resources required to process and analyze imagery at the rate it can be collected. The rapid development of unmanned underwater vehicles suggests researchers will soon have access to an even greater volume of imagery and other sensor measurements than can be collected by diver-piloted platforms, further exacerbating data handling limitations. Thoroughly segmenting (tracing the extent of and taxonomically identifying) organisms enables researchers to extract the information image products contain, but is very time-consuming. Analytic techniques driven by neural networks offer the possibility that the segmentation process can be greatly accelerated through automation. In this study, we examine the efficacy of automated segmentation on three different image-derived data products: 3D models, and 2D and 2.5D orthographic projections thereof; we also contrast their relative accessibility and utility to different avenues of biological inquiry. The variety of network architectures and parameters tested performed similarly, ∼80% IoU for the genus *Porites*, suggesting that the primary limitations to an automated workflow are 1) the current capabilities of neural network technology, and 2) consistency and quality control in image product collection and human training/testing dataset generation.

## 1 Introduction

Unmanned remote sensing platforms offer the prospect of collecting for monitoring and analysis vastly more image-based data than was previously possible. In particular, the high-resolution imagery made available by the IKONOS and MODIS satellites revolutionized macroecological studies across terrestrial habitats (Pfeifer et al., 2012). In the marine sciences, these technologies have enabled observation of the entirety of the planet’s oceans regularly. However, the opacity of water prevents similarly comprehensive viewing of the ocean floor beyond the shallowest areas. Capabilities of unmanned underwater vehicles (UAVs) for closer, finer-scaled inspection are not as well-developed as satellites but are advancing rapidly. In shallow, nearshore environments like coral reefs, ecological research groups are increasingly transitioning away from diver-recorded data to manned underwater imagery surveys—analogous to what a UAV would collect—that can be archived and analyzed (Weinberg, 1981; Gracias and Santos-Victor, 2000).

Using structure from motion (SfM) algorithms, 3D models of reef tracts can be derived from survey imagery (Pizarro et al., 2009; Smith et al., 2016; Edwards et al., 2017; Ferrari et al., 2017; Kodera et al., 2020; Sandin et al., 2020), enabling analysis in a data medium that much more closely resembles real-world coral environments, particularly in steep or geometrically-complex areas that are common in reefs but poorly captured with top-down imagery. A variety of metrics, including percent cover, growth, species composition, or disease or bleaching incidence can be extracted from 3D models, or 2D orthographic projections (orthoprojections) thereof, using random point sampling or full semantic segmentation (i.e., labeling per-point, per-taxa; hereafter referred to simply as segmentation). Image-based data increases the amount of information collected from a field site relative to *in situ* observation approaches, but it introduces new data extraction challenges: it is much simpler to amass a library of thousands of reef sites than it is to extract the ecological information contained in that library.

Neural networks and other computational/algorithmic processing and analytical strategies offer hope that such backlogs can be cleared. A number of studies have evaluated automated segmentation methods, both neural-network-based and otherwise, for classifying (applying a label to an entire scene) or segmenting (applying labels per-pixel) 2D images of coral (Beijbom et al., 2012) or orthoprojections of 3D models of coral reefs (Alonso et al., 2017, 2019; Alonso and Murillo, 2018; Yuval et al., 2021). Beyond coral applications, neural networks for top-down photographic media have been tested in 2D, 2.5D (RGBD), 3D, and hybrid formats in contexts such as satellite imagery with promising results (Mohammadi et al., 2019; Bachhofner et al., 2020; Saralioglu and Gungor, 2020; Song and Choi, 2020). More generically, many studies have examined 2D, 3D, and hybrid methods of segmentation, with the two most common applications being 1) scene analysis to inform automated decision-making for driverless cars, grasping arms, and other robotic applications, and 2) rapid or assisted interpretation of diagnostic medical imagery. Development in these fields is quite rapid, but as of this date no automated solution exists to segment corals with the accuracy desired by coral scientists hoping to reliably track growth on the scale of, in some cases, mere millimeters per year.

In this study, we compare the performance of 2D, 2.5D, and 3D segmentation neural networks on 10 m × 10 m reef pointclouds, and 2D and 2.5D orthoprojections thereof, from Palmyra Atoll in the Pacific. We aim to contribute to the effort of evaluating the utility of neural-network-expedited segmentation workflows, as well as mapping out scenarios where inexpensive and fast 2D analysis is adequate and those where benefits of 3D are substantial.

## 2 Materials and Methods

### 2.1 Photographic Survey, 3D Model and 2D Orthoprojection Derivation

The details of our field sampling design have been discussed in detail elsewhere (Edwards et al., 2017; Fox et al., 2019; Kodera et al., 2020; Sandin et al., 2020). Briefly, the photographic surveys were conducted by a pair of divers within 100 m^2^ plots positioned along the 10m isobath in oceanic fore reef habitats. Utilizing a custom-built frame containing two Nikon d7000 DSLR cameras mounted in tandem, the diver operating the camera collected imagery *via* a lawnmower pattern 1.5 m above the site, with cameras set to a 1 s interval timer and oriented straight down in the direction of gravity (as opposed to perpendicular to the surface plane of the reef). The first camera was equipped with a wide angle 18 mm lens to ensure the substantial overlap required for accurate 3D model reconstruction, while the second was equipped with a 55 mm lens to provide the visual detail needed to disambiguate complex species-level identifications. During each survey, a series of scale bars and boundary markers were placed throughout the plot, which are visible in the final reconstructed model. At each plot boundary marker, a second diver recorded depth information to establish orientation of the plot relative to the plane of the ocean surface.

The details of the technical processing software used to generate the 3D models have been described previously (Westoby et al., 2012; Sandin et al., 2020). Briefly, the models used in this study were created with Photoscan, now known as Metashape, which is developed by Agisoft LLC. Scale bars and depth measurements were used as ground control points to determine scale and orientation. The resulting model is a pointcloud: a list of points with XYZ spatial coordinates and corresponding RGB color values.

Pointcloud visualization and geometric analysis in this study was performed in the software package Viscore, developed at UC San Diego in the Cultural Heritage Engineering Initiative/Kuester Lab (Petrovic et al., 2014; Fox et al., 2019; Sandin et al., 2020). Viscore is a visual analysis platform that facilitates a number of 3D workflows useful for performing virtual fieldwork, including interactive alignment and inspection of time-series site representations, manual plot segmentation in 3D, import/export/filtering of point subsets, and generation of orthoprojected maps and digital elevation models.

### 2.2 Dataset

The models studied in this report were collected annually from 2013 to 2020 on Palmyra Atoll at a single 10 m × 10 m site. Top-down 2D orthoprojections were derived for each 3D model at a scale of 1 mm/pixel. Corresponding 2.5D versions of the orthoprojections were produced by including the Z depth value for each pixel. All *Porites* (a coral genus) within the study site were traced by hand in Viscore. Therefore, every point/pixel had one of two possible labels: *Porites* or everything else. For ingestion into neural networks, 3D models were split into 1 m × 1 m patches along the *X* and *Y* axes (defined as the plane of constant depth), while orthoprojections were split into image patches 512 pixels on a side. This resulted in 100 1 m^2^ 3D patches and 400 512 × 512 pixel patches per year. Each 512^2^ pixel patch corresponds to approximately 0.25 m^2^. These dimensions were chosen because they had roughly similar memory impact in our experiments, resulting in the use of equivalent batch size (8) during training across all dimensionalities. 60% of patches from years 2013–2019 (420 pointcloud patches, 1,680 image patches) were randomly assigned to the training subset, while another 20% each was reserved for validation and testing. To test how the neural network models would perform on a model no part of which was in the training set, the entire 2020 model was reserved for separate testing.

Performance on data collected under different circumstances than that which is in the training set is a critical metric when trying to determine how useful an automated tool is. This concept is known as generalization. It is analogous to the difference between memorization and conceptual understanding—tools that generalize well perform consistently across different times, places, and contexts, while those that generalize poorly only perform well on inputs that closely resemble examples they have already seen. In the context of this study, a neural network that generalizes well would accurately segment *Porites* photographed anywhere in the world, with any camera, at any time of day, etc., because it has developed an understanding of how to distinguish *Porites*, while one that generalizes poorly would only segment accurately when presented with *Porites* from our single site on Palmyra between the years 2013 and 2019 because it has memorized how to make correct predictions in that specific context. Automated tools that generalize well are much more useful than those that do not.

*Porites* in each 10 m × 10 m plot were manually segmented in Viscore by one of the authors (NP, CE, HR, or EA). Viscore offers high-framerate 3D rotation and zooming, survey image draping, and point labeling with adjustable-size paintbrushes to make the manual segmentation process as easy and accurate as possible. Which author segmented which plot is shown in Table 1. The segmenters are separated into two categories: those with extensive coral identification experience (“experts”), and those with less (“non-experts”). All four of the authors also segmented the same 3 m × 3 m subsection of the 2020 plot to evaluate consistency between ourselves.

**TABLE 1:** Coral segmenters, their experience, the plot(s) they traced, and IoU on the 3 m × 3 m subsection of the 2020 model relative to consensus.

Tracer	Experience	Years traced	IoU v. consensus on 2020 subsection [%]
Hugh Runyan (HR)	Non-expert	2013, 2014, 2018, 2019, 2020	88.6
Clint Edwards (CE)	Expert	2016	93.7
Nicole Pedersen (NP)	Expert	2015	94.1
Esmeralda Alcantar (AE)	Expert	2017	92.4
-			Mean: 92.2

### 2.3 Segmentation With Neural Networks

Unsegmented 3D pointclouds and derived 2D orthoprojections can be used to investigate a variety of characteristics of ecological importance, such as 3D reef structure and qualitative observations of coral colony health. However, far more information is available if each organism is identified and segmented. Repeated surveys of a location depict the recruitment, growth, and death of individual corals, so they can be used to evaluate competition, successional states, equilibrium, anthropogenic impacts, and more. Manually segmenting a single square meter requires an average of 1 hour; at this rate, an expert working full time would need at least a century to map every organism in the ∼2000 pointclouds thus far collected by the research labs contributing to this report. Expediting segmentation is therefore necessary to make full use of photographic survey products. Neural networks are the state-of-the-art solution for segmentation of visual media such as images, videos, and 3D models.

For this study, we tested SparseConvNet (SCN), a successful point cloud segmentation neural network architecture (Graham et al., 2018). SCN does not operate directly on pointclouds, but instead voxelizes (the 3D equivalent of a pixel) the input pointcloud to a chosen grid size. Voxels with side length 2 mm were used for this study. Smaller voxels increased computation time but did not improve predictions. 3D computations are done sparsely, meaning on a list of occupied voxels instead of as matrix operations on every location whether it is occupied or not. Sparse operations greatly improve speed and efficiency operations on data in which most matrix locations are empty, which is the case with our reef surface; there is no useful information above or below the surface, so a 3D cube containing that surface is mostly empty space. SCN is fast, simple, and provides the additional benefit of operating on discretized locations, just as the 2 and 2.5D image segmentation neural networks we used in this study do. For 2.5D segmentation we used ESANet (Seichter et al., 2021), while 2D segmentation was done with ESANet with its depth channel components disabled (in this configuration it only considers RGB color information, as opposed to color and depth). ESANet uses a ResNet-34 (He et al., 2015) encoder with an additional “context module,” similar to those found in the popular DeepLab architectures (Chen et al., 2018), for detecting large-scale patterns. These architectures were chosen because they perform well on publicly-available benchmarks and are among the more commonly used.

All networks were trained with batch size 8 for 512 epochs with the Adam optimizer (Kingma and Ba, 2015) and the cross entropy loss function, using similar color, scale, and deformation augmentations. During training, the networks were evaluated with the 2013–2019 validation set and saved at regular intervals (every 10 epochs), after which the best performing of the saved versions were selected and then tested on the 2013–2019 and 2020 test sets. The results of these tests are shown in Table 2.

**TABLE 2:** Highest automated segmentation accuracy of each neural network dimensionality as determined by validation IoU score.

	*Porites* IoU (2013–2019 validation set) [%]	*Porites* IoU (2013–2019 test set) [%]	*Porites* IoU (2020) [%]
2D	82.8	82.0	57.4
2.5D (RGBD)	83.1	81.4	45.0
3D	81.4	82.3	70.4

The difference between 2.5D and 3D neural networks is subtle. 2.5D neural networks operate in flat, two-dimensional windows placed over an image; using, say, a 5 × 5 window of pixels, the network searches for patterns in each of the RGB color channels, as well as the Z depth channel, and combinations thereof. In a 3D neural network, the analogous window would be 5 × 5 × 5. Patterns of color are sought in different 3D shapes, just as in 2D neural networks patterns of color are sought in 2D shapes. The primary shortcoming of the 2.5D approach is that each pixel can only contain one value for each of R, G, B, and Z—if two surfaces share the same horizontal location in XY, only the one with the higher Z value will be stored in an RGBD image.

### 2.4 Error Metrics

For training we used the cross-entropy loss function. To evaluate predicted segmentations, we use the intersection over union (IoU) error metric. For each class (meaning possible labels, here just 1) *Porites* and 2) everything else), IoU is defined as the intersection of the prediction and ground truth—the true positive count—divided by the union of the prediction and ground truth—the sum of the true positive, false positive, and false negative counts: TP/(TP + FP + FN).

In contrast, accuracy is defined as (TP + TN)/(TP + TN + FP + FN). Because prediction is relatively easy for most of the ∼95% of our plots that is not *Porites*, TN will be a very large number relative to TP for the *Porites* class. If the automated segmentation network identified 5% of the plot as *Porites* but it was entirely the wrong 5%, it would still be 90% accurate, because only 10% of the points are mislabeled (5% that should have been labeled *Porites* but weren’t and 5% that should not have but were). This means that segmentation accuracy is a less useful metric for performance evaluation than IoU; IoU better reflects our goal of tracking the growing edges of individual corals of specific genera that often comprise only a small fraction of the total area of the plot.

## 3 Results and Discussion

### 3.1 Consistency Amongst Labelers

**FIGURE 1:**
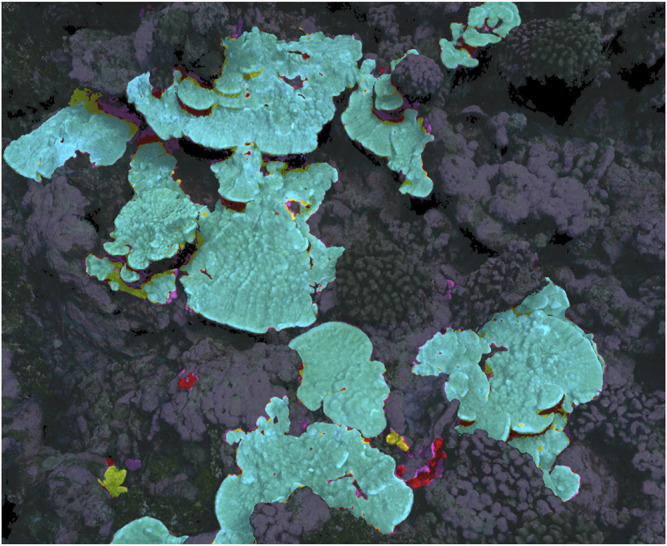
3D model showing agreement and disagreement amongst manual segmenters. Dark background is consensus background, while blue highlights show agreed-upon *Porites* by all four segmenters. Points highlighted yellow were labeled *Porites* by three segmenters, red by two, and purple by only one.

**FIGURE 2:**
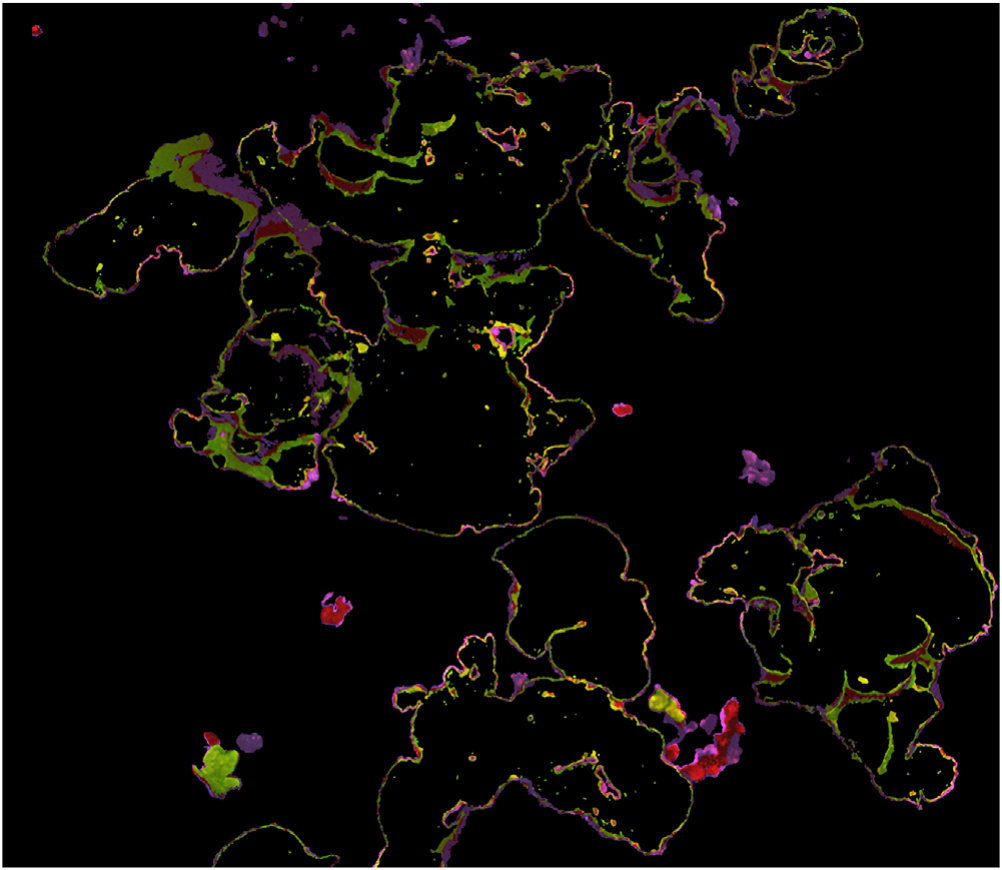
3D model showing manual segmenter *Porites* disagreement only. Points highlighted yellow were labeled *Porites* by three of four segmenters, red by two, and purple by only one.

All four authors that segmented the same 3 m × 3 m subsection of the 2020 plot agreed on 76% of points that were labeled *Porites* by at least one author, meaning that, of the points labeled *Porites* by at least one segmenter, 76% of them were labeled *Porites* by all four. These segmentations are illustrated in Figure 1, while the disagreed-upon points are isolated in Figure 2. This bulk disagreement metric is not ideal however, as it is likely to be influenced by the number of segmenters (more people will disagree more often than fewer people). We created a more stable alternate accuracy metric by first creating a “ground truth” from majority consensus labeled points (those labeled *Porites* by at least three of us), and then calculated IoU error against that ground truth for each labeler. Results are shown in Table 1. The mean IoU of all labelers against majority consensus ground truth was 92%, with the non-expert scoring the lowest at 88%.

### 3.2 Automated Segmentation

**FIGURE 3:**
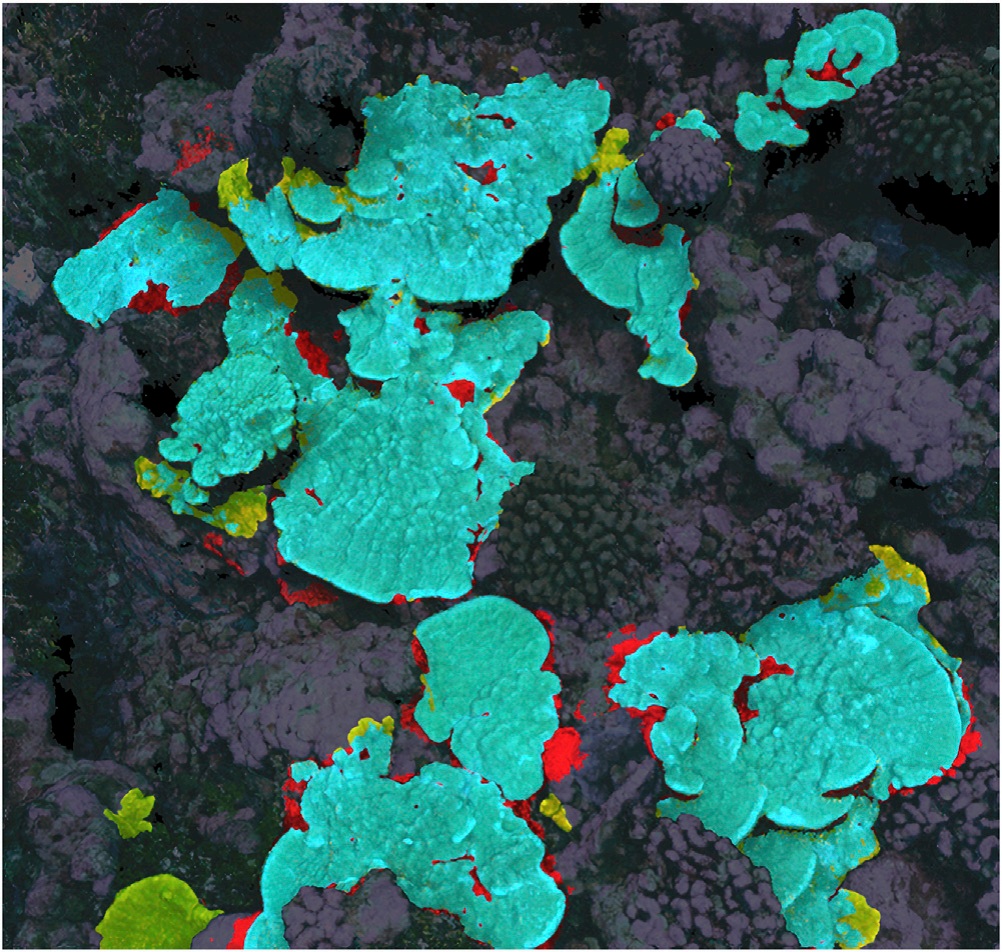
Example of 3D neural network prediction (correct predictions are highlighted blue, false positive predictions are red, and false negative are yellow), corresponding to the same area as Figures 1, 2.

Prediction IoU scores for the top-performing 2D, 2.5D, and 3D neural networks as determined by validation IoU score are reported numerically in Table 2, while a visual example of 3D results is shown in Figure 3, corresponding to the same area of the 2020 model that was used for the consistency experiment in Section 3.1. 2D, 2.5D, and 3D approaches performed similarly (∼80% IoU) on the 2013–2019 validation and test sets, but performance on the 2020 plot held out of the training dataset varied: the 3D network achieved the highest 2020 score (70.4%), while the 2D network scored second highest (57.4%), and the 2.5D network scored the lowest (45.0%).

Prediction IoU scores on the 2013–2019 test set of the most successful 3D network (as determined by validation IoU) are broken down by year in Table 3. Scores were relatively stable, ranging from 77.2% to 85.6%. Scores on the 2015, 2016, and 2017 models traced by experts were 84.6%, 85.6%, and 77.2% respectively, while scores on non-expert-traced models were 78.5%, 83.1%, 85.3%, and 84.9%.

**TABLE 3:** Automated segmentation accuracy of the 3D network on the 2013‐2019 test set broken down by year.

Year	*Porites* IoU (test set) [%]
2013	78.5
2014	83.1
2015	84.6
2016	85.6
2017	77.2
2018	85.3
2019	84.9

The consistency of performance across media dimensionality is likely explained by a combination of factors. First and foremost, our 2D and 2.5D orthoprojections are derived directly from the 3D model, which is constructed from top-down survey imagery. As such, there is little in the 3D models that is not in the orthoprojections because images were not captured from side-on angles. While the extra dimensions offer additional opportunities for pattern detection, if there is little difference in significant information between the three input dimensionalities, then there are few resulting additional patterns for the higher-dimensional networks to detect. A second important factor is that these 2D, 2.5D, and 3D neural networks are algorithmically quite similar: they all depend on cascaded multi-scale convolutional pattern detectors. Fundamentally, neural networks are optimization problems just like curve-fitting, and as in that simpler scenario there are often a number of similarly-performing algorithmic alternatives but none that are near-perfect without overfitting.

A third factor to consider is labeling errors and subjective decisions, in both the training set and the ground truth used to evaluate performance—it is possible that the accuracy/consistency of our *Porites* training and testing dataset is only ∼80%. If there are errors in training data, a neural network might fail to learn useful information or learn incorrect information, while errors in the validation and test sets introduce uncertainty into performance metrics. As can be seen in Figures 1–3, much of both segmenter disagreement and neural network error concerned exact placement of coral colony boundaries. If training data is inconsistent in those areas, the neural network may not be able to learn how to be precise and consistent when delineating boundaries, and if the validation/test data is inconsistent in those areas it may be difficult to tell from performance metrics like IoU if the network is right or wrong.

The comparison between contributing segmenters (Section 3.1, Table 1) suggested individual segmenters score ∼90% by IoU when compared to a more reliable consensus segmentation. However, that experiment was only conducted on one small 3 m × 3 m patch of reef where segmenters knew they would be compared to one another. It is possible the segmenters were fatigued or less careful when segmenting entire 10 m × 10 m models and made more mistakes, especially as four of the seven models were segmented by the least-experienced contributor (HR). If the 2013, 2014, 2018, and 2019 segmentations produced by the least-experienced author (HR) were indeed more flawed, we might expect the neural network IoU prediction scores on those years to be lower, but the per-year results in Table 3 show that automated performance was relatively consistent across years, and the lowest score (77.2%) occurred on a model traced by one of the experts.

The better performance of the 3D network on the 2020 plot, none of which was in the training set, could be because, while color varies due to e.g. camera settings and lighting conditions, 3D structure is more consistent across samples. If this explanation were correct, we would expect the 2.5D network to outperform 2D on the 2020 dataset because 2.5D also contains information about physical structure, but that was not the case (Table 2). It is possible that the 3D networks are also structurally smaller and simpler than 2D or 2.5D networks because the latter are more memory efficient and developmentally mature. More complex networks often perform better, but sometimes become more sensitive to small deviations in data distribution (generalize more poorly).

Generally speaking, these results should not be expected to consistently extrapolate to any coral dataset from anywhere on the globe. This is especially true of performance on the 2020 dataset. Neural networks’ accuracy depend on a number of factors, such as: the number of genera they are asked to predict and the phenotypic plasticity of those genera, variation in lighting conditions during photo surveys, including turbidity and depth-dependent color changes, specifics of the camera sensor and settings including resolution, white balance, focal length, manual or autofocus settings, as well as the image acquisition pattern, vagaries of the 3D reconstruction process, and more. As shown by the relatively good and consistent performance of all three dimensionalities on the 2013–2019 validation and test sets, neural networks can learn to account for these variations if they are present in the training dataset—if training and testing sets strongly resemble one another. The variable and unreliable performance on the 2020 dataset demonstrates a commonly-understood shortcoming of neural networks: they often don’t perform well on samples from outside the dataset on which they were trained. IoU scores on the 2020 dataset were dramatically lower, even though the 2020 dataset was from the same geographic site on Palmyra as 2013–2019 data (i.e., the corals were mostly the same, excepting a year’s worth of growth and death) and all datasets were collected with similar equipment and procedures. Many strategies have been developed on how to limit performance loss on unseen data, generally referred to as domain adaptation or domain generalization, but they are beyond the scope of this report.

Coral researchers hoping to expedite segmentation with neural networks should strive for consistency, comprehensiveness, and quality control in field image collection methodology and manual segmentation of training and validation data, but the results in this report suggest automated performance loss is likely in applications concerning unsegmented new survey sites or resurveys of existing sites.

For most ecological applications, the neural network prediction accuracy achieved in this study is not adequate, so researchers must manually correct the automated predictions before they can be used in analysis. Such human-in-the-loop workflows (Pavoni et al., 2021) are more realistic at this time than fully automated ones, and they have the added advantage of a human expert verifying the segmentations as they are created. Relying on automated predictions carries the risk of the accuracy of those predictions changing without researcher detection, resulting in mislabeled data and erroneous conclusions. However, this manual editing and verification process is time-consuming: in our experience, the time required to segment an entire plot *de novo* and the time required to edit predictions is often similar. Generally, the most time-consuming aspect of manual segmentation is tracing complex boundaries. Unfortunately, as illustrated in Figure 3, that is the area neural network predictions tend to be least reliable, so boundaries often must be segmented manually whether one is editing imperfect predictions or segmenting *de novo*. Further, Figures 1, 2 show that boundaries are also the place manual segmenters are most likely to disagree. The boundaries of coral segmentations can be viewed as zones of uncertainty, where neither humans nor neural networks are reliable. Researchers intending to use segmentations of image products to study mm-scale growth at the boundaries of individual coral colonies are advised to rigorously engage this topic.

### 3.3 Considerations of Data Dimensionality

While it is a well-vetted analytical approach, in many applications working in 2D has significant shortcomings—largely arising from a 2D model’s inherent limitations in fully representing a 3D environment. Sites exhibiting greater geometric complexity—with ledges and overhangs, or with branching coral morphologies (e.g. *Acropora*)—cannot be fully represented in either 2D or 2.5D. Aspects of 3D structure unaffected by occlusion—colony height, say, or benthic slope or rugosity—are representable in 2.5D but not in 2D. Another more subtle issue arises concerning the direction of orthoprojection: the pattern of occlusions, as well as the relative density at which different parts of a site are (re)sampled (for gridded models such as DEMs) both depend on the chosen projection direction. Furthermore, 2D measurements—distances, projected areas, angles, (non-detrended) rugosity estimates—are all affected by the choice of projection. Selecting the projection direction systematically and consistently is especially important for making quantitative comparisons (e.g. growth rates) across time series. Orthoprojecting along the direction of gravity is often a reasonable standard—however, many reefs contain habitats of interest that feature steep slopes, or stark vertical structures, especially on shallow reef flats and forereefs, both of which are critical habitats for study. In such cases, orthoprojecting along the direction of gravity makes little sense as the projected area of organisms in these habitats will represent a fraction of their true footprint in 3D. Measuring the exact vertical direction underwater can also be difficult and imprecise, which introduces projection error—particularly undesirable when trying to accurately measure a few millimeters of growth at colony edges.

However, working in 3D presents its own set of challenges, making greater demands than 2D on every stage of the workflow, from acquisition to analysis to dissemination. Full 3D datasets are much larger than 2D—orthoprojections range from 0.5–2 GB per time point, with depths per pixel adding another ∼1 GB, while the 3D models in this study range from 10 GB to 75 GB per time point—and require significantly more computational power to work with. This technical challenge is addressed by the Viscore platform, allowing multi-year multi-site projects (easily totaling several TB of data) to be inspected and annotated interactively on commodity laptops.

Even with data-handling impediments removed, working in 3D is generally more difficult than in 2D. Reconstructing complete 3D models (without holes) requires more photographs, from more view-angles, than are required for 2D. In this work, we photograph primarily with the camera pointed straight down: the resulting models are incomplete underneath overhangs or on vertical surfaces where sight lines are occluded. The peripheries of images, especially from wide-angle lenses, capture much of the information necessary to build accurate digital surrogates, but they nevertheless do not capture the entirety of the 3D structure of the reef.

This incompleteness of 3D coverage complicates analysis. Measuring colony size and growth is one example: while top-down projected areas can be reliably measured in 2D (subject to the 2D caveats discussed above), making similar measurements in 3D (surface area and volume) requires more care. Measuring total 3D surface area is relatively straightforward for well-defined surfaces, but models resulting from incomplete coverage (e.g., only top-down views) require a degree of assumption or interpolation in areas of low coverage and reconstruction point density. Further, estimates of volume can be particularly problematic, as for many coral taxa they require assumptions of where the coral skeleton ends and the underlying substrate begins—information often not captured by top-down imagery—though changes in volume can be more reliably estimated. Scale and model resolution also have a significant impact on 3D measures: coral often has much more 3D surface area than 2D (particularly branching, corymbose, and foliose forms), due to structures ranging from m- or cm-sale (e.g., branches) to sub-mm corallite walls, ridges, and other delicate features. Accurately capturing 3D structure at such small scales is difficult. In an ideal world the coral research community would work exclusively with complete sub-mm resolution 3D models, but that is not always practical.

Those coral researchers pursuing applications where the drawbacks of 2D are relatively minimal (e.g., where the morphology of the taxa of interest is not overly complex, the bathymetry is relatively flat, the most useful information is contained in color rather than 3D morphology) will likely prefer 2D due to speed and simplicity. On the other hand, those more concerned with geometric fidelity in complex environments will prefer to operate with 3D pointclouds if the necessary resources can be mustered.

## Data Availability

The raw data supporting the conclusion of this article will be made available by the authors, without undue reservation.
